# Amputation and gangrene associated with SGLT2 inhibitors: pharmacovigilance analysis of the FDA adverse event reporting system (FAERS) database

**DOI:** 10.1007/s40618-026-02809-3

**Published:** 2026-01-08

**Authors:** Alyaa M. Ajabnoor

**Affiliations:** 1 Department of Pharmacy Practice, Faculty of Pharmacy, King Abdulaziz University, P.O. BOX 80324, Jeddah, 21589 Saudi Arabia; 2 Division of Pharmacy and Optometry, School of Health Sciences, Faculty of Biology, Medicine and Health, University of Manchester, Manchester, UK

**Keywords:** SGLT2 inhibitors, Adverse events, FAERS, Amputation, Fournier’s gangrene

## Abstract

**Background:**

Sodium-glucose co-transporter-2 (SGLT2) inhibitors are widely prescribed to diabetic patients for their cardiorenal benefits. However, concerns over adverse events—specifically amputation risk and gangrene—have risen in recent years. This study aimed to describe the frequency and severity of these events in relation to SGLT2 inhibitor use, using the U.S. FDA Adverse Event Reporting System (FAERS).

**Methods:**

OpenVigil 2.1 was used to extract safety reports submitted to FAERS from 2003 Q4 to 2024 Q4. Amputation and gangrene events linked to SGLT2 inhibitors (canagliflozin, dapagliflozin, empagliflozin, and ertugliflozin) were identified and compared to other drugs. Reporting odds ratios (RORs) were calculated.

**Results:**

A total of 3,540 adverse event reports were identified: 2,128 for canagliflozin, 361 for dapagliflozin, 1,049 for empagliflozin, and 2 for ertugliflozin. Canagliflozin was strongly associated with toe amputation (ROR = 635.6; 95%CI: 566.7–712.1), foot amputation (ROR = 201.8; 95%CI: 174.3–233.7), and Fournier’s gangrene (ROR = 117.5; 95%CI: 100.0–138.0). Empagliflozin was linked with Fournier’s gangrene (ROR = 565.2; 95%CI: 525.3–608.2) and toe amputation (ROR = 21.2; 95%CI: 17.6–25.6). Dapagliflozin showed weaker signals; ertugliflozin had minimal data.

**Conclusion:**

Toe amputation and Fournier’s gangrene were most reported with canagliflozin and empagliflozin, reinforcing prior safety concerns and the need for monitoring.

## Introduction

Sodium-glucose co-transporter-2 inhibitors (SGLT2is) is a class of oral hypoglycemic drugs that is relatively new, first approved by the US Food and Drug Administration (FDA) for the management of type 2 diabetes in 2013. Since the approval of SGLT2is, they have been widely prescribed for patients with type 2 diabetes mellitus due to their ability to reduce glycosylated hemoglobin, lower blood pressure, and reduce weight [1]. They have also showed superiority when compared to placebo in slowing the progression of chronic kidney disease, heart failure, and Atherosclerotic Cardiovascular Disease (ASCVD) [2–4]. More recently, accumulating evidence has shown that SGLT2 inhibitors exert important anti-inflammatory and immunomodulatory effects, including reductions in proinflammatory cytokines and downregulation of inflammatory immune cell activity, which may contribute to their cardio-renal protective benefits [5]. 

Several safety communication have been issued by the FDA concerning SGLT2is such as serious infection of the genitals, [6] risk of acute kidney injury, [7] serious urinary tract infections, [8] and increased risk of bone fractures [9]. Following post-marketing safety observations and findings from the Canagliflozin and Cardiovascular and Renal Events in Type 2 Diabetes (CANVAS) trial—which evaluated the cardiovascular effects of canagliflozin and identified a nearly twofold increase in the risk of lower-limb amputation—the concern regarding SGLT2is intensified [3]. This serious adverse event led to regulatory warnings by the U.K. Medicines and Healthcare Products Regulatory Agency (MHRA) [10] and the U.S. FDA issuing a Boxed warning in 2017 [11]. Later in 2019 findings from the CREDENCE [12] trial showed that canagliflozin have both cardiovascular and renal protective effects in patients with type 2 diabetes. Based on that, the FDA have removed the Boxed warning since the overall benefits of canagliflozin outweighed the potential amputation risk and included a precautionary warning in the prescribing information [13]. Thus, the objective of this study is to utilize the United States Food and Drug Administration Adverse Event Reporting System (FAERS) to understand more about the frequency and the severity of these adverse events (AEs) in relation to SGLT2 inhibitors use. Being aware of these events can play a crucial role in enhancing the overall treatment efficacy and a decrease in morbidity for diabetic patients.

## Methods

### Study design

This was a retrospective pharmacovigilance study that included reports of gangrene and lower limb complications among patients treated with SGLT2 inhibitors in the U.S. FAERS database. FAERS was established by the FDA as part of its post marketing safety surveillance program for drugs. It is a well-organized spontaneous reporting database that contains AEs resulting from reports of medication errors, adverse drug reactions , or product quality complaints. FAERS receive global reports from healthcare professionals, consumers, and drug companies. FAERS currently includes data from the fourth quarter (Q4) of 2003 to 2024 Q4. OpenVigil version 2.1 was used to extract data from FAERS [14]. OpenVigil is an AEs data extraction and cleaning, mining, and analysis tool specifically designed for the FAERS database, it utilizes the U.S. Adopted Name (USAN) system to categorize and identify medications. USAN offers standardized and official nonproprietary names for drugs (e.g., “canagliflozin” rather than the brand name “Invokana”). Since reports of AEs frequently include free-text drug names (which may have typographical errors, abbreviations, or brand names), only those reports in which the drug name could be distinctly and accurately matched to a USAN name were incorporated into the analysis. This approach ensures that the data is accurate and trustworthy, reducing the likelihood of errors stemming from naming confusion. In this study, cases were included if they were defined as AE reports, in which the reporter referred to an SGLT2 inhibitor (canagliflozin, empagliflozin, dapagliflozin, ertugliflozin, ipragliflozin, tofogliflozin, luseogliflozin, remogliflozin, and their combination with other drugs) as a “Primary Suspect.” AEs were classified and described according to the preferred terms (PTs) and the system organ classes (SOCs) in the international MedDRA, version 24.0. Individual case safety reports (ICSRs) included in the study had to contain an SGLT2 inhibitor reported as a primary suspect and an AE term reporting at least one of the following events (Fournier’s gangrene, cellulitis gangrenous, diabetic gangrene, gas gangrene, dry gangrene, amputation, leg amputation, foot amputation, limb amputation, toe amputation, diabetic foot, or osteomyelitis). Since FAERS database contains numerous updates on cases, which means an entire case may include many unique reports, only entire cases were used for analysis, therefore, a case contributes to the result if at least one of its reports includes the SGLT2 inhibitor-event relationship.

### Statistical analysis

Disproportionality analysis was performed using OpenVigil 2.1. Data was extracted for AEs associated with SGLT2 inhibitors , and all reports up to 2024 Q4 were included. Although, OpenVigil 2.1 currently have data from 2003 Q4, only data from March 29, 2013 was relevant to this study since canagliflozin was first approved by the US. FDA in 2013. The primary measure of disproportionality was the Reporting Odds Ratio (ROR), calculated using a 2 × 2 contingency table: (a) the number of reports of a specific AE with the drug, (b) the number of reports without the AE with the drug, (c) the number of reports of the AE for all other drugs, and (d) the number of reports without the AE for all other drugs. A signal was considered statistically significant if the ROR > 2.00 and the lower bound of the 95% confidence interval (CI) exceeded 1.00. Analyses were restricted to reports where the SGLT2 inhibitor was identified as the primary suspect drug. OpenVigil automatically removes duplicate entries, corrects formatting errors, and standardizes drug names to ensure data consistency. Since data from FAERS are publicly available, no ethics committee approval was required for this study.

## Results

**Fig. 1:**
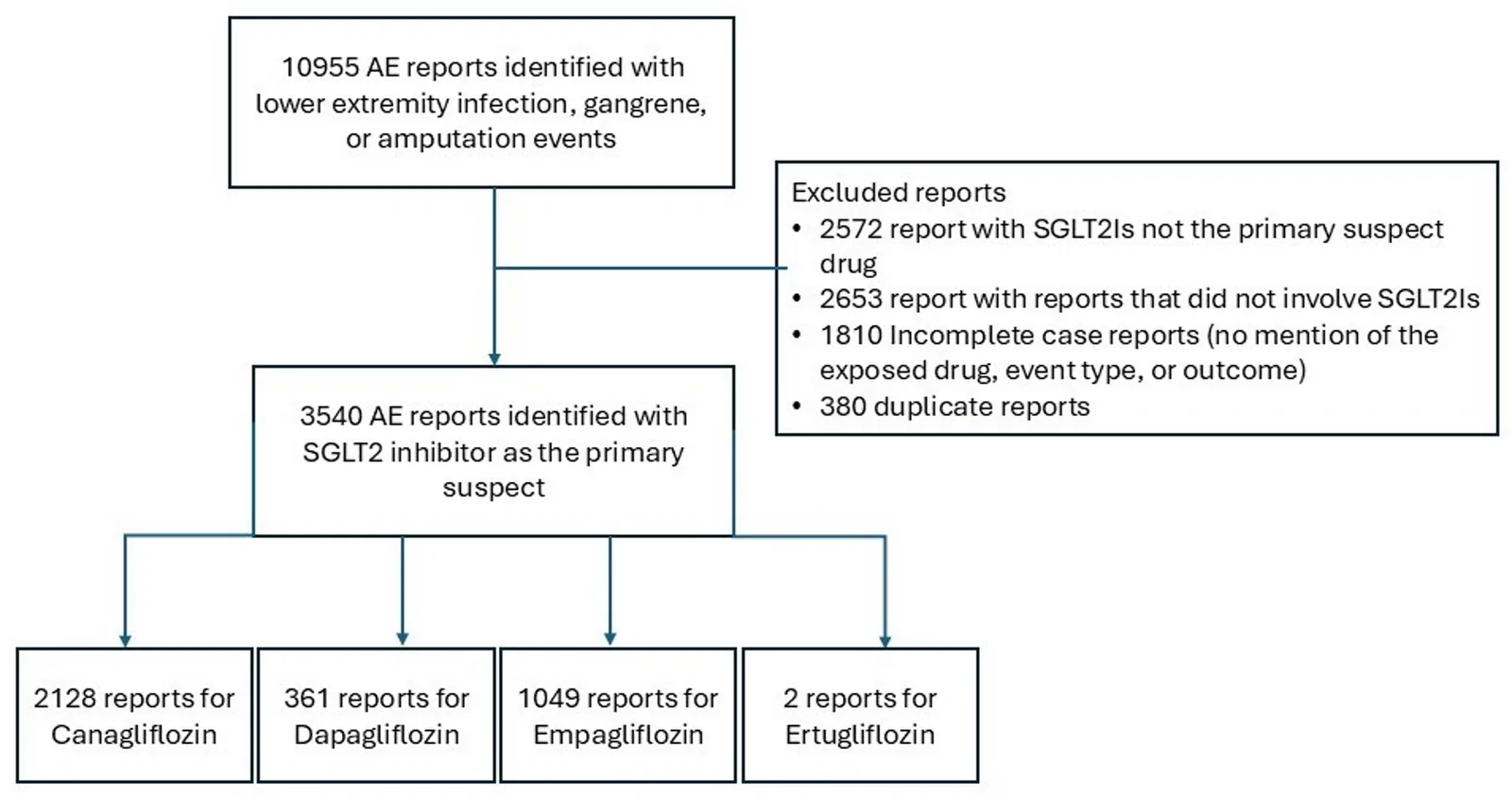
Cases from FAERS database meeting inclusion criteria for review. AE: Adverse Event, SGLT2is: Sodium-glucose co-transporter-2 inhibitors

**Fig. 2:**
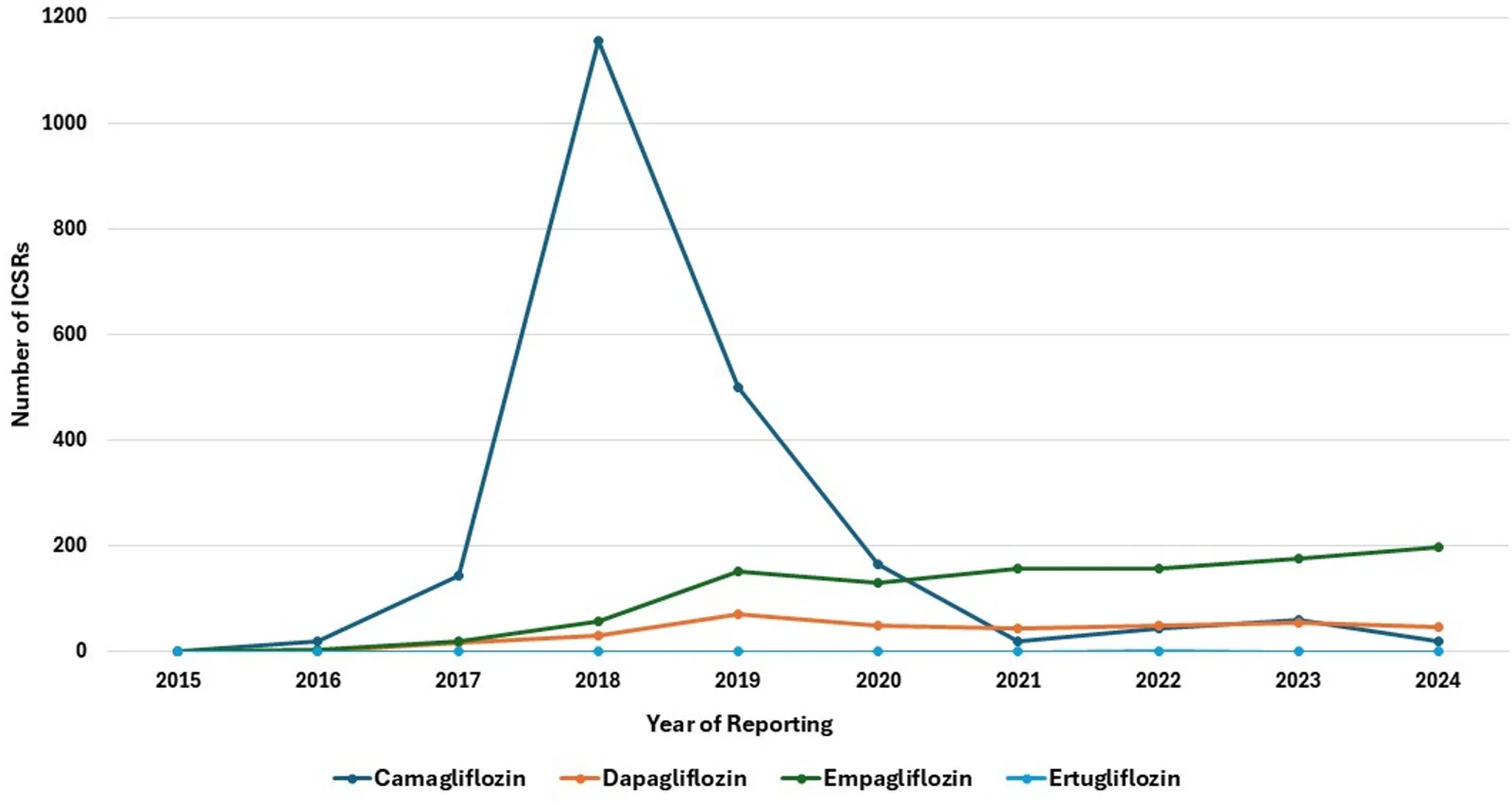
Number of adverse event reports of SGLT2 inhibitors from the United States

In the FAERS database, from 2003 Q4 to 2024 Q4, a total of 10,955 adverse event reports involving amputation or gangrene events associated with SGLT2 inhibitors were identified (Fig. 1). After removing duplicates and incomplete reports a total of 3540 reports were found. Of these, 2,128 reports were linked to canagliflozin, 361 to dapagliflozin, 1,049 to empagliflozin, and only two cases were linked to ertugliflozin, reflecting minimal reporting activity. The majority of reports involved male patients representing 67.5% to 100% of cases, with a mean age of patients ranging from 56 years for canagliflozin to 61 years for empagliflozin indicating that events were predominantly reported in older adults (Table 1). Among the reported outcomes, hospitalization accounted for approximately 40% of all cases. Disability was also recorded in approximately 20% of canagliflozin-related reports, 14% of dapagliflozin-related reports, and 12% of empagliflozin-related reports. Death was more commonly reported with dapagliflozin (6.7%) and empagliflozin (4.4%). The majority of reports came from North America particularly for canagliflozin (95.7%). While reports from European countries contributed a significant portion of reports for dapagliflozin (32.0%) and empagliflozin (32.0%). Overall, reporting of events reached its peak in 2018 for canagliflozin with 1155 cases (54.3%) then declined steadily in the following years (Fig. 2). As for reports for empagliflozin it reached its maximum in 2024 with 197 reports (18.8%), while dapagliflozin had its highest number of reports in 2019 with 70 cases (19.4%). The event counts in Table 1 exceed the total number of unique reports because FAERS allows multiple adverse events to be recorded for a single case. Therefore, multiple complications (e.g., diabetic foot with concurrent osteomyelitis or different types of amputation) can be reported for the same individual case. For canagliflozin, the most frequently reported event was ‘amputation of no specific type’ (89.6%). However, among specific types, toe amputation was the most common, reported in 50.3% of cases. Fournier’s gangrene was the most reported event for both dapagliflozin (60.4%) and empagliflozin (74.5%) (Table 1).

**Table 1:** Patient demographics and adverse event characteristics of lower limb complications and necrotizing conditions reports associated with SGLT2 inhibitors

Characteristics	Canagliflozin	Dapagliflozin	Empagliflozin	Ertugliflozin
Number of cases	2128	361	1049	2
Male (%)	1437 (67.5)	245 (67.9)	698 (66.5)	2 (100)
Mean age	56 (9)	59 (12)	61 (11)	Not specified
Outcome (%)				
Hospitalization-initial or prolonged	1054 (49.5)	140 (38.8)	422 (40.2)	0
Life-threatening	29 (1.4)	41 (11.4)	121 (11.5)	0
Disability	427 (20.1)	51 (14.1)	126 (12.1)	0
Death	27 (1.2)	24 (6.7)	46 (4.4)	0
Other medical events	591 (27.8)	105 (29)	334 (31.8)	2 (100)
Reporting country (%)				
North America	2041 (95.7)	198 (52.0)	561 (51.8)	2 (100.0)
Europe	58 (2.7)	122 (32.0)	346 (32.0)	0
Asia	29 (1.4)	36 (9.4)	73 (6.7)	0
Oceania	2 (0.1)	16 (4.2)	89 (8.2)	0
South America	1 (0.0)	8 (2.1)	11 (1.0)	0
Africa	0 (0.0)	1 (0.3)	2 (0.2)	0
Unknown	2 (0.1)	0 (0.0)	0 (0.0)	0
Year of report (%)				
2015	1 (0.0)	0 (0.0)	0 (0.0)	0
2016	19 (0.9)	1 (0.3)	3 (0.3)	0
2017	144 (6.8)	16 (4.4)	20 (1.9)	0
2018	1155 (54.3)	30 (8.3)	57 (5.4)	0
2019	500 (23.5)	70 (19.4)	151 (14.4)	0
2020	165 (7.8)	48 (13.3)	130 (12.4)	0
2021	20 (0.9)	44 (12.2)	158 (15.1)	0
2022	43 (2.0)	49 (13.6)	156 (14.9)	2 (100.0)
2023	61 (2.9)	56 (15.5)	177 (16.9)	0
2024	20 (0.9)	47 (13.0)	197 (18.8)	0
Event (%)*				
Fournier’s gangrene	159 (7.5)	218 (60.4)	781 (74.5)	0
Cellulitis gangrenous	5 (0.2)	1 (0.28)	3 (0.3)	0
Diabetic gangrene	19 (0.9)	2 (0.28)	4 (0.3)	0
Amputation (no specific type)	1907 (89.6)	119 (33)	211 (20.1)	2 (100)
Leg amputation	414 (19.4)	27 (7.5)	50 (4.8)	0
Foot amputation	284 (13.3)	19 (5.3)	24 (2.3)	0
Limb amputation	104 (4.9)	6 (1.7)	14 (1.3)	0
Toe amputation	1071 (50.3)	58 (16.1)	104 (9.9)	2 (100)
Diabetic foot	607 (28.5)	17 (4.7)	39 (3.7)	2 (100)
Dry gangrene	50 (2.4)	5 (1.4)	5 (0.5)	2 (100)
Gas gangrene	52 (2.4)	0	3 (0.3)	0
Osteomyelitis	1125 (52.9)	16 (4.4)	44 (4.2)	0

*Frequencies may exceed total number of unique reports due to co-occurrence of multiple events per case

### FAERS database from 2015 to 2024. ICSRs: individual case safety reports

**Fig. 3:**
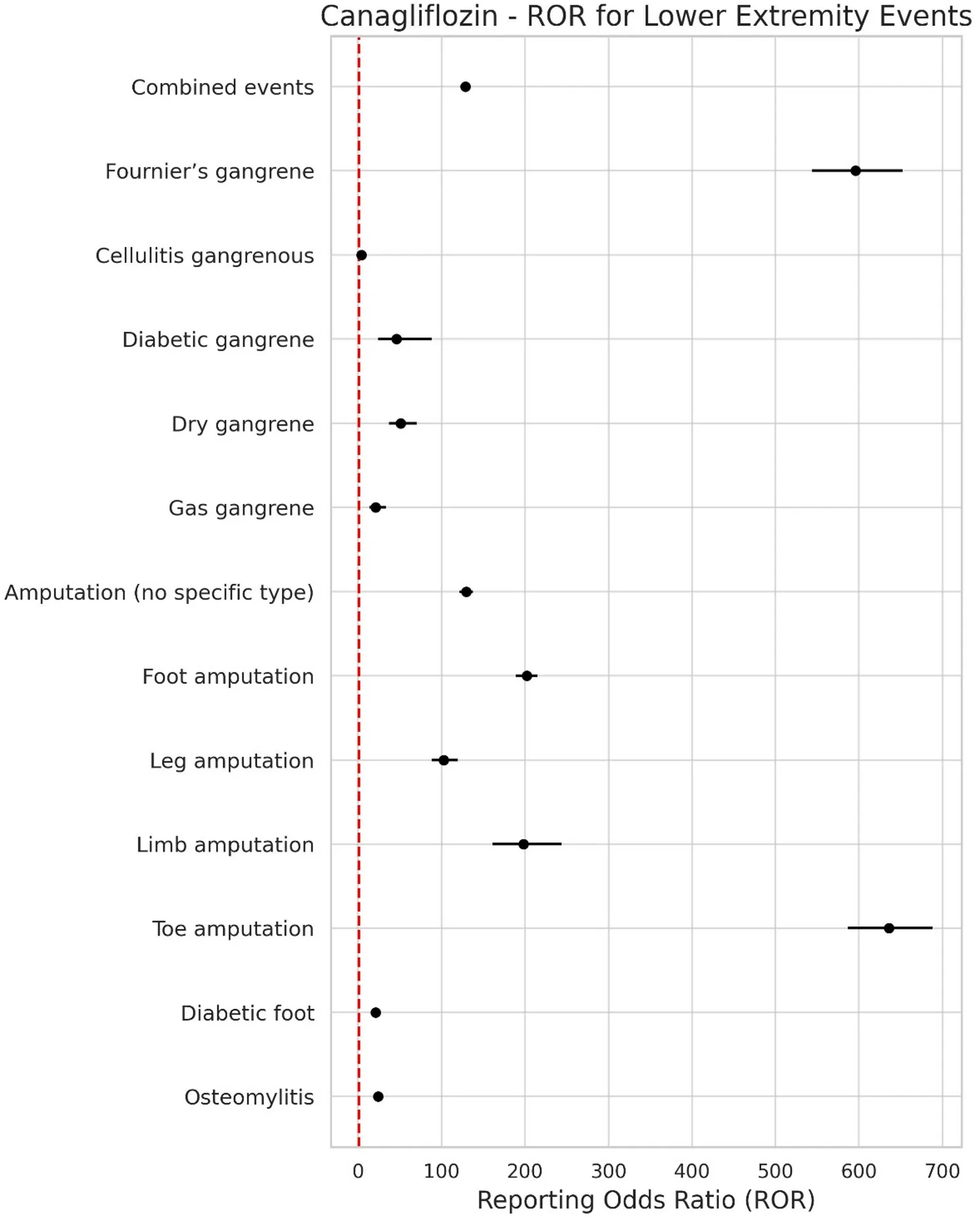
Reporting Odds Ratios (RORs) for adverse events associated with canagliflozin use in FAERS (2015–2024)

**Fig. 4:**
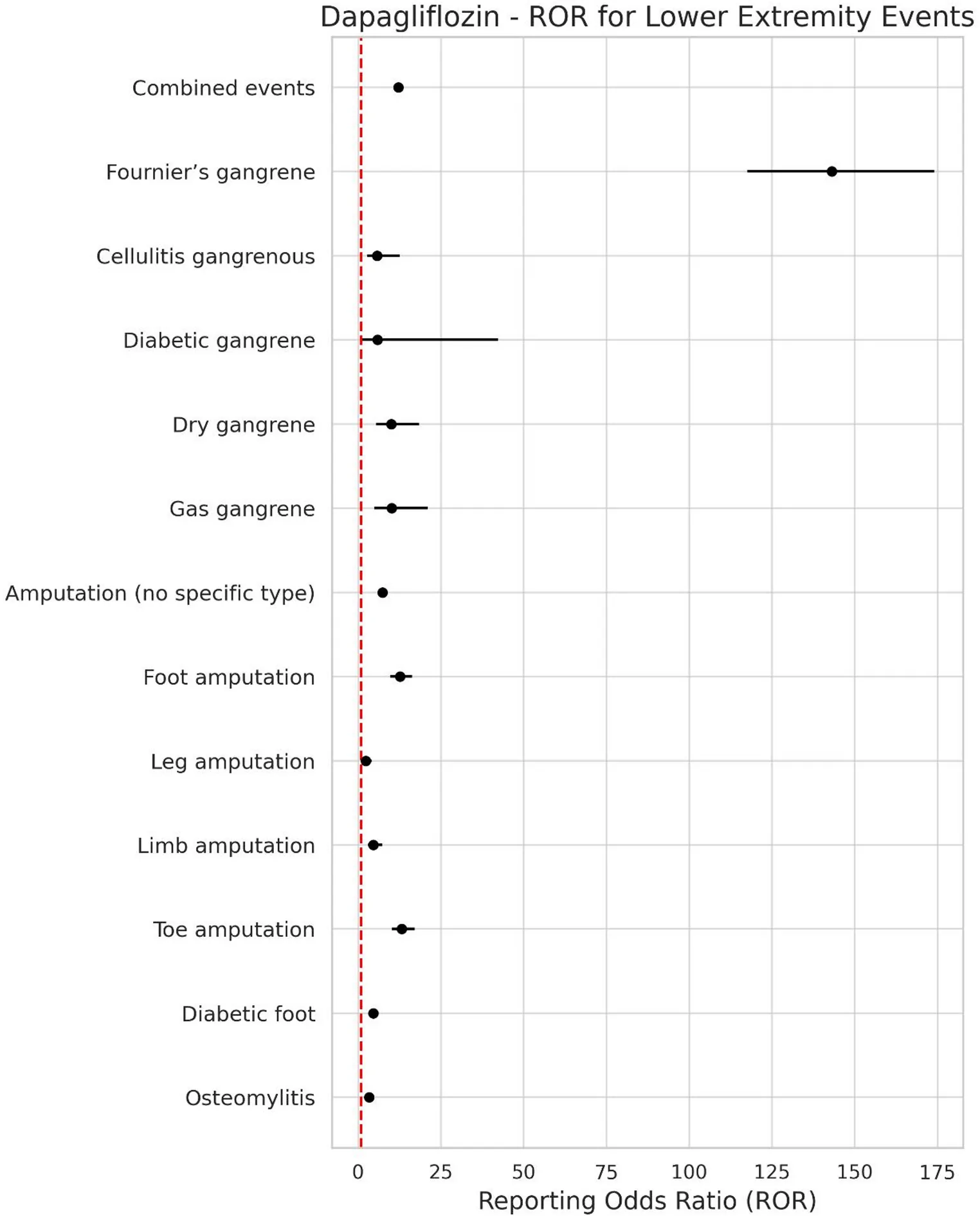
Reporting Odds Ratios (RORs) for adverse events associated with dapagliflozin use in FAERS (2015–2024)

**Fig. 5:**
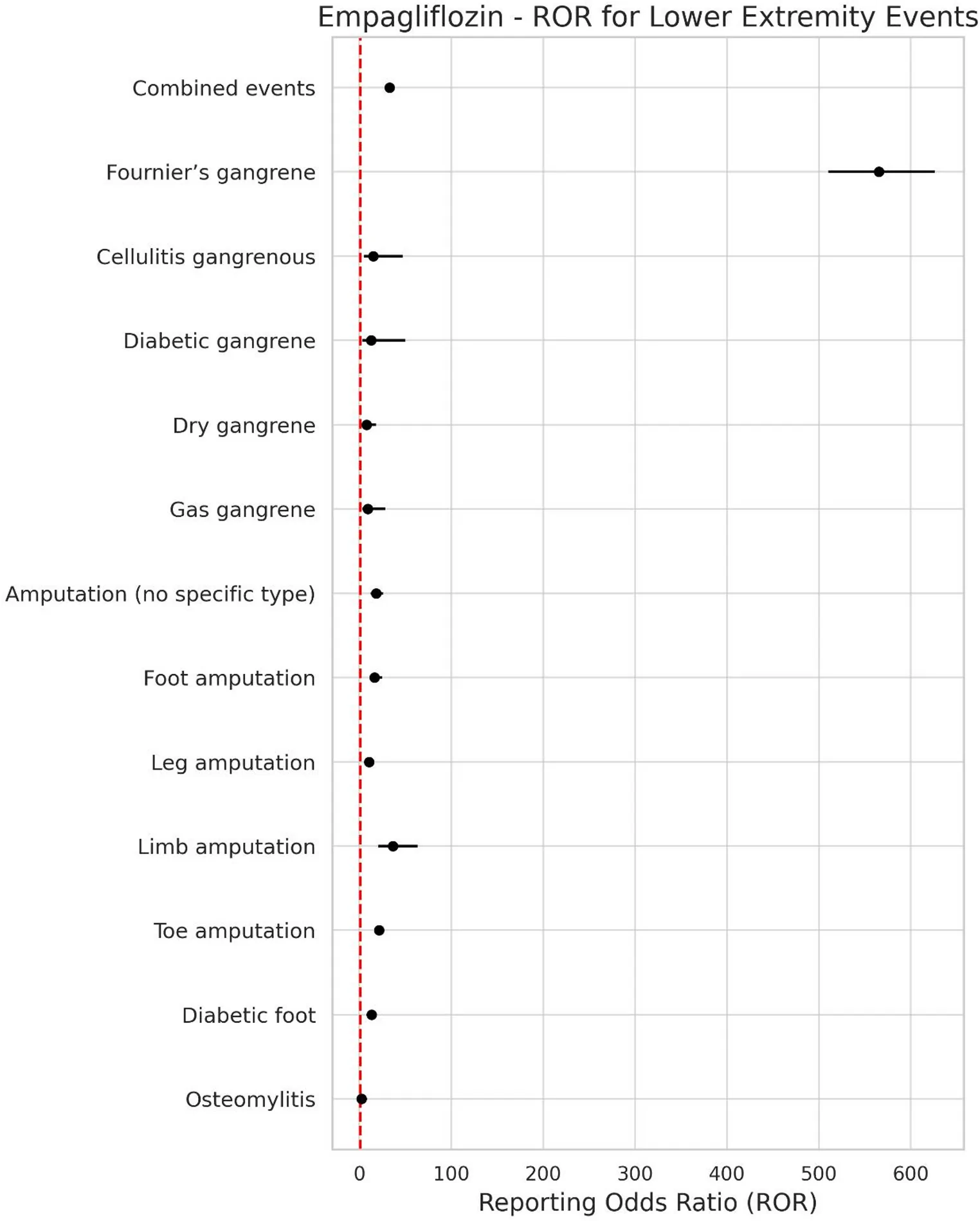
Reporting Odds Ratios (RORs) for adverse events associated with empagliflozin use in FAERS (2015–2024)

The forest plots illustrate the reporting odds ratios (RORs) and 95% confidence intervals for various lower extremity adverse events associated with canagliflozin, dapagliflozin, and empagliflozin (Figs. 3, 4 and 5). Overall, canagliflozin demonstrated the strongest disproportionality signals, with notably elevated RORs for toe amputation (ROR: 635.63; 95% CI: 586.76–688.56), Fournier’s gangrene (ROR: 595.68; 95% CI: 543.86–652.49), and unspecified amputation (ROR: 129.33; 95% CI: 121.38–137.79) (Fig. 3). Dapagliflozin also exhibited significant signals, particularly for Fournier’s gangrene (ROR: 143.03; 95% CI: 117.56–174.01) and toe amputation (ROR: 13.15; 95% CI: 10.13–17.06) (Fig. 4), though at lower magnitudes compared to canagliflozin (Fig. 2). Empagliflozin showed elevated RORs for Fournier’s gangrene (ROR: 565.21; 95% CI: 509.98–626.42), limb amputation (ROR: 36.08; 95% CI: 20.54–63.37), and toe amputation (ROR: 21.19; 95% CI: 17.42–25.77) (Fig. 5), indicating relevant safety signals despite fewer overall cases.

## Discussion

This pharmacovigilance study utilized data from the FAERS database to investigate the association between SGLT2 inhibitors and lower extremity complications, and necrotizing conditions The findings demonstrated marked differences in the RORs for adverse events among canagliflozin, dapagliflozin, empagliflozin, and ertugliflozin. Canagliflozin and empagliflozin were particularly associated with the highest RORs for combined adverse events, specifically toe amputation and Fournier’s gangrene reinforcing prior safety concerns raised by regulatory bodies and randomized controlled trials.

The CANVAS trial first brought attention to the increased risk of lower limb amputation with canagliflozin compared to placebo report a Hazard Ratio (HR) of 1.97(95% CI: 1.41–2.75) that lead to issuing a warning for canagliflozin from both the European Medicines Agency and the US.FDA [3]. The FDA warning for lower limb amputation risk was issued in 2017, and for Fournier’s gangrene was issued in 2018 [6]. Our analysis showed that the number of reports for canagliflozin peaked in 2018 and continued to decline till 2024, and reports for empagliflozin started in 2017 then gradually peaked in 2024. The decline trend in reporting both amputation risk and Fournier’s gangrene can be a result of healthcare professionals adopting a more cautious approach when prescribing canagliflozin and considering factors that may predispose patients to the need for amputations or Fournier’s gangrene. Such as peripheral vascular disease, neuropathy, diabetic foot ulcers, symptoms of tenderness, redness, or swelling of the genitals. Empagliflozin was not originally linked to increased risk of amputation risk and Fournier’s gangrene following EMPA-REG trial [4]. However, the rise in the number of adverse events reports for empagliflozin after 2017 in this analysis may reflect the expanded use of empagliflozin following EMPA-REG results, [4] and greater post-market awareness after issuing the canagliflozin warning.

In this analysis, despite the lower RORs associated with dapagliflozin compared to canagliflozin and empagliflozin, it did exhibit a positive signal related to serious adverse events with Fournier’s gangrene and toe amputation. These findings are in line with previous reports of safety signals with SGLT2 inhibitors. Chang et al. analyzed the FAERS database and observed potential risks with several SGLT2 inhibitors beyond canagliflozin, including dapagliflozin, although with lower magnitude [15]. Similarly, another study by Sato et al. using data from the Japanese Adverse Drug Event Report (JADER) database have found signals linking dapagliflozin to serious lower limb complications. However, results from the DERIVE trial found no significant association between dapagliflozin and increased risk of amputation, [16] which indicates that the observed signals in spontaneous reporting systems may not be generalizable across different populations. These contrasting findings underscore the variability in outcomes across different study designs, data sources, and patient populations. Notably, ertugliflozin showed only two reported cases and no detectable disproportionality signal. Although the number is insufficient for meaningful interpretation, reporting ertugliflozin alongside other SGLT2 inhibitors provides a complete class-wide pharmacovigilance profile. The minimal number of reports may reflect lower market uptake, shorter post-marketing exposure time, or differences in real-world reporting practices.

The exact mechanism of SGLT2 inhibitors induced risk of lower limb complications is not entirely understood and remains multifactorial. Verma et al. [17] emphasized on the renal and cardiovascular benefits associated with SGLT2 inhibitors, however, there seems to be some theoretical concerns related to their vascular effects in certain patients especially those with predisposing risks to peripheral ischemia. In their review they discussed how SGLT2 inhibitors can cause hemoconcentration and volume depletion secondary to their osmotic diuretic effect which could impair microvascular perfusion and worsen pre-existing peripheral vascular disease. Verma et al. [17] emphasized that while SGLT2 inhibitors confer robust cardiovascular and renal benefits, there are theoretical concerns regarding their vascular effects in certain populations, these hemodynamic changes may heighten susceptibility to ischemic complications in the lower extremities, especially in patients with underlying vascular compromise. This FAERS-based findings support this mechanistic concern, as amputation, osteomyelitis, and diabetic foot complications were frequently co-reported with canagliflozin and empagliflozin use—agents known to induce pronounced diuretic effects. Therefore, clinicians should exercise caution when prescribing SGLT2 inhibitors to individuals with impaired limb perfusion and consider regular foot examinations and early vascular assessments during treatment. Although, recent data from genetic-instrument using Mendelian randomization analyses suggest no significant causal association between SGLT2 inhibition and most lower-limb safety outcomes (e.g. osteomyelitis, ulcers, cellulitis), though a potential risk for peripheral arterial disease remains uncertain [18]. Similarly, a 2024 multicenter cohort study comparing SGLT2 inhibitors with DPP-4 inhibitors reported no increased below-knee amputation risk in real-world practice [19]. These findings highlight the ongoing debate and underscore that disproportionality signals from spontaneous reporting systems like FAERS should be interpreted cautiously and considered hypothesis-generating rather than definitive evidence of causal risk.

Fournier’s gangrene is a rare but necrotizing soft tissue infection that involves the perineum and external genitalia [20]. It is considered a surgical emergency and often requires urgent debridement and intensive management. In this FAERS-based analysis, empagliflozin had the highest number of reports for Fournier’s gangrene followed by canagliflozin, and dapagliflozin, indicating a potential class-wide effect with variable magnitude. These trends align with a previous pharmacovigilance evaluation from the FAERS database, which demonstrated disproportionate reporting of Fournier’s gangrene in association with SGLT2 inhibitors, especially canagliflozin [21]. It was previously reported that although Fournier’s gangrene is rare, it appeared more frequently in SGLT2 inhibitor users than in patients treated with other antidiabetic agents, with canagliflozin and empagliflozin being most frequently implicated [22]. However, a recent study by Stottlemyer et al. [23] using FAERS data up to March 2022, that assessed the disproportionality of adverse events related to SGLT2 inhibitors found that only canagliflozin was significantly associated with amputation and osteomyelitis, while no such signal was observed with other SGLT2 inhibitors such as empagliflozin and dapagliflozin. Our analysis, which extended to the end of 2023 and considered a wider set of outcomes including Fournier’s gangrene, observed signals not only with canagliflozin but also with empagliflozin and dapagliflozin. This broader signal profile may reflect increased usage of newer SGLT2 inhibitors over time and a growing recognition of associated adverse events post-2017 regulatory alerts. In contrast to these pharmacovigilance findings, a Cochrane systematic review of randomized controlled trials concluded that SGLT2 inhibitors probably have little or no effect on the risk of amputation compared to placebo [24]. Nonetheless, unlike spontaneous reporting systems like FAERS, randomized trials may not capture rare or delayed adverse events, particularly in high-risk populations. Therefore, real-world data from pharmacovigilance systems remain critical to signal detection and post-marketing safety surveillance, particularly for rare but serious complications like Fournier’s gangrene. Our study adds further weight to this signal, underscoring the importance of vigilance when prescribing SGLT2 inhibitors, particularly in individuals with risk factors such as poorly controlled diabetes or immunosuppression.

This study has several limitations inherent to pharmacovigilance analyses using spontaneous reporting systems such as FAERS. First, the data is subject to underreporting and reporting biases, including stimulated reporting following regulatory alerts or media attention. Second, the FAERS database lacks denominator data, such as the total number of drug exposures, precluding incidence rate estimation or risk quantification. Third, this study is constrained by the limited data available through OpenVigil, which only includes basic variables such as age, sex, drug, event, and outcome. Access to detailed clinical information like comorbidities, laboratory values, or cardiovascular history was limited. Also, incomplete or inconsistent reporting in FAERS further limits the ability to interpret causality. These constraints prevent multivariable analysis and exploration of mechanisms like microvascular inflammation. Moreover, reported patterns reflect database trends and do not establish causal links to serious outcomes such as hospitalization, disability, or amputation. Fourth, OpenVigil categorizes event outcomes (e.g., hospitalization, disability) using fixed fields, which reflect reporting trends rather than definitive clinical endpoints. Overall, these limitations highlight the need to interpret findings as hypothesis-generating signals that warrant further investigation through controlled studies and real-world evidence.

## Conclusion

This pharmacovigilance analysis of FAERS data identified a disproportionate number of reports for lower extremity complications, particularly amputations and Fournier’s gangrene—associated with certain SGLT2 inhibitors, especially canagliflozin and empagliflozin. While these findings align with prior safety signals and regulatory warnings, they must be interpreted cautiously due to the inherent limitations of spontaneous reporting systems, including underreporting, lack of clinical details, and the inability to establish causality. Nonetheless, these signals are clinically relevant and highlight the need for continued post-marketing surveillance and prescriber vigilance, especially in patients with predisposing risk factors for peripheral ischemia or soft tissue infections.

## Data Availability

The data is available through the OpenVigil website https://openvigil.sourceforge.net/.
